# Comparative Phyloclimatic Analysis and Evolution of Ecological Niches in the Scimitar Babblers (Aves: Timaliidae: *Pomatorhinus*)

**DOI:** 10.1371/journal.pone.0055629

**Published:** 2013-02-06

**Authors:** Árpád S. Nyári, Sushma Reddy

**Affiliations:** 1 Department of Biology, Loyola University Chicago, Chicago, Illinois, United States of America; 2 Department of Zoology, Oklahoma State University, Stillwater, Oklahoma, United States of America; Michigan State University, United States of America

## Abstract

We present the first extensive and integrative analysis of niche evolution based on climatic variables and a dated molecular phylogeny of a heterogeneous avian group of Southeast Asian scimitar babblers of the genus *Pomatorhinus*. The four main clades of scimitar babblers have species that co-occur in similar areas across southern Asia but some have diverged at different timeframes, with the most recently evolved clade harboring the highest number of species. Ecological niche models and analysis of contributing variables within a phylogenetic framework indicate instances of convergent evolution of members of different clades onto similar ecological parameter space, as well as divergent evolution of members from within clades. *Pomatorhinus* species from different clades occupying Himalayan foothills show convergence towards similar climatic tolerances, whereas within a clade, allopatric sister-species occurring in the Himalayas have diverged to occupy different climatic parameter spaces. Comparisons of climatic tolerances of Himalayan foothills taxa with species distributed further south in Assam/Burma and Burma/Thailand indicate convergence towards similar parameter spaces in several climatic variables. Niche overlap was observed to be lower among species of the youngest clade (*ruficollis*) and higher among species of older clades (*ferruginosus*). Analysis of accumulation of ecological disparity through time indicates rapid divergence within recent time frames. As a result, Himalayan taxa originating at different temporal scales within the four main scimitar babbler clades have differentiated ecologically only in recently diverged taxa. Our study suggests that the repeated orogenic and climatic fluctuations of the Pliocene and Pleistocene within mainland Southeast Asia served as an important ecological speciation driver within scimitar babblers, by providing opportunities for rapid geographic expansion and filling of novel environmental niches.

## Introduction

Biogeography is at the interface between historical and ecological explanations for species distributions. One of the fundamental questions in biogeography is, how do species accumulate in different regions? Historical interpretations examine speciation patterns driven by processes such as vicariance and dispersal, while ecological explanations include environmental tolerance and niche partitioning. Here we aim to address this critical question by integrating both historical and ecological inferences to examine the evolution of environmental niche occupancy using speciation patterns across different clades.

Development of environmental niche modeling tools has led to numerous studies examining various aspects of species distributions including ecological determinants and historical limits of species distributions [1]–[5]. Ecological niche models integrate the study of climatic tolerances through combining species occurrence data and environmental variables over the species’ past, present and future geographic range. Although some studies have examined temporal variation in species distributions [6], [7], only few have examined niche evolution within clades using phylogenetic history – an emerging field called phyloclimatic analysis [8]–[13]. In this study, we used phyloclimatic analysis in a comparative context to determine ecological niche evolution across four separate but related clades of songbirds endemic to tropical Asia. Our main objectives were to examine how species in different clades come to occupy the same areas and evolve similar habitat requirements and contrast the ecologies of species colonizing novel areas.

Despite harboring one of the highest species diversities in the world, the avifauna of tropical Asia is only now being examined using modern phylogenetic tools. One of the main consistent findings across phylogenetic studies is that taxonomy is egregiously erroneous, both in terms of representing the accurate numbers of distinct evolutionary lineages within what has been regarded a single species [14]–[17] and the designation of novel families, genera and species due to reclassification of related groups [18]–[24]. With the accumulation of phylogenetic analyses of Asian groups, we can begin to ask more synthetic questions of how this biota was assembled.

**Figure 1:**
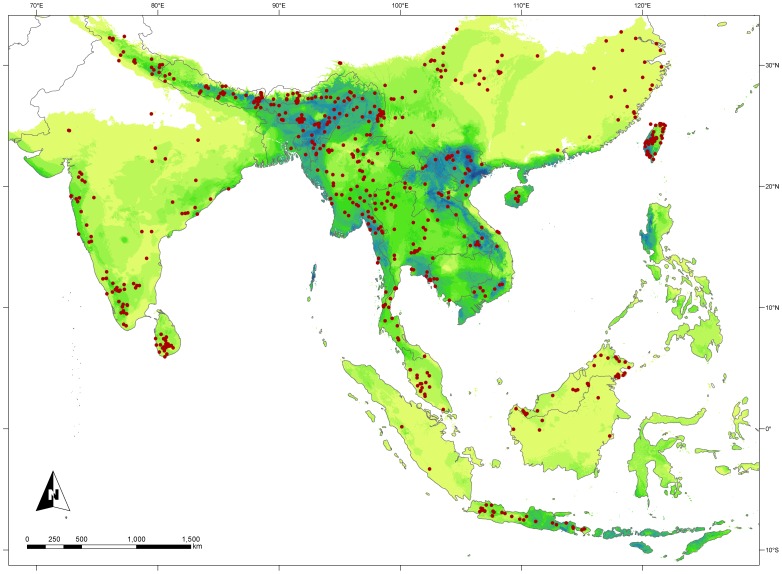
Map illustrating the geographic region of *Pomatorhinus* scimitar babblers. Sampled occurrence points used in ecological niche model analyses are indicated by red dots. Overlap of resulted ecological niche models of the 29 species included in the study ranges from highest species density indicated in blue hues (maximum of 14 species), while areas of single species occurrence are denoted in light green. Note that disjunct geographic areas such as the islands of the Philippines and Sulawesi do offer climatic conditions suitable for scimitar babblers, although no taxa presently occur in these areas. See also Figure S1 for a summary of ecological niche models of each of the 29 *Pomatorhinus* species.

**Figure 2:**
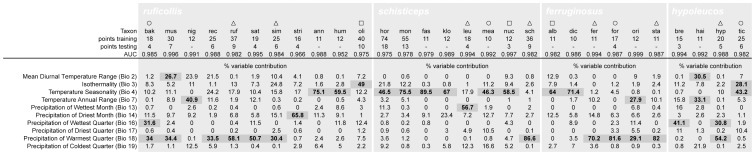
Summary of input data and MAXENT model parameters. Summary of number of occurrence points and MAXENT modeling algorithm variable contributions and model AUC scores for 29 species of Southeast Asian *Pomatorhinus* scimitar babblers. For taxa with 10–12 occurrence points, a cross-validation sampling has been employed in MAXENT and averages are being reported throughout the table. Taxa are grouped by clades identified through the phylogenetic analysis outlined in Figure 3. Species abbreviations reflect the first three letters of their specific epithet, and taxa annotated by open triangles occupy Himalayan foothills, while open circles and open squares denote species occupying highlands at lower latitudes (Assam/Burma and Burma/Thailand, respectively). Variable abbreviations are given in brackets and are referenced throughout the main text and the supplementary materials. Variable contributions to overall model performance greater than 25% are indicated in bold and darkened boxes.

The avian family Timaliidae, or babblers, is a highly diverse group with its concentration of diversity centered in southern Asia [24], [25]. Babblers are not only rich in numbers of species but also in morphological, ecological, and behavioral diversity. In terms of biogeography, babblers are interesting in that many species are co-distributed in areas of endemism across tropical Asia. The scimitar babblers (Timaliidae: *Pomatorhinus*) are typical members of the family in terms of morphology, behavior, and geographic distribution [25]. A recent molecular phylogenetic study of the traditionally defined clades of scimitar babblers identified novel interrelationships leading to the recognition of 4 distinct clades with as many as 27 phylogenetic species [17] ranging from the lowlands, foothills and montane forests of the Indian subcontinent to southern China, Southeast Asia, and the Greater Sundas (Figure 1, Figure 2). These related and co-distributed lineages span homologous biogeographic areas and present an ideal group for investigating the ecological niche breadth and climatic tolerances of member species in a phylogenetic context.

Of particular interest in this group and its four main clades is a general biogeographic split between species currently distributed in the Sino-Himalayan regions (i.e. Himalayas, Burma, Yunnan) and Southeast Asian taxa (i.e. Vietnam, Thailand, Cambodia, Malay Peninsula), which highlights the ecological opportunity offered by the uplift of the Tibetan plateau and adjacent highlands throughout southern Asia [20], [26]–[28]. Among its constituent clades, species of the *ruficollis* group have witnessed more extensive and recent cladogenesis compared to those in the other 3 clades (*hypoleucos, ferruginosus* and *schisticeps*). Moreover, Himalayan species in the *ruficollis* and *schisticeps* clades diverged more recently (ranging from 0.12–0.16 and 0.27–0.42–0.58 Mya, respectively) compared to species in the *hypoleucos* and *ferruginosus* clades (ranging from 2.7–3.8 and 3.0–4.1 Mya, respectively) [17]. These similarities and disparities in geographic breaks and timing of divergence between the four *Pomatorhinus* clades highlight salient features of a highly adaptable radiation within continental Southeast Asia.

Given the variable prevalence of ecological niche conservatism across temporal scales [29]–[32], incorporating underlying ecological information in a phylogenetic context will prove insightful into the deciphering of historical processes and climatic tolerance limits that act as potential drivers or constraints of diversification at various spatio-temporal scales. Only few such integrative studies have recently been directed towards groups of organisms from continental Asia [33], [34], and none have provided a comparative analytical framework using phyloclimatic modeling [1], [2], [12], [13] of Southeast Asian vertebrate taxa. We therefore make use of the combination of dated molecular phylogenetic information for the *Pomatorhinus* scimitar babblers [17] and ecological niche models [35]–[37] based on georeferenced occurrence data and bioclimatic variables summarizing the ecological landscape of the group’s native range to 1) characterize the abiotic niche space of *Pomatorhinus* scimitar babblers, 2) quantify niche occupancy (breadth, overlap, and disparity) within and between the 4 distinct clades, 3) investigate the history of ecological niche occupancy and accumulation of disparity through time, and 4) compare the niche signatures of Himalayan taxa to species distributed further south in the highlands of Assam/Burma and Burma/Thailand. These questions are meant to address the need of quantifying and comparing the evolutionary stability of ecological niche characteristics over phylogenetic history, allowing insights into the diversification of songbirds within Southeast Asia.

## Methods

Two main sources of data are necessary for the analysis of ecological characteristics in a phylogenetic context. First, a phylogenetic hypothesis in the form of a topology with branch lengths and divergence times derived through the analysis of molecular characters of extant *Pomatorhinus* species; and second, ecological niche models, estimated via species occurrence points and environmental data from the Grinellian niche, focusing solely on characterizing climatic tolerances throughout a species’ range [38].

### Molecular Phylogenetic Analysis and Dating

We used the molecular dataset based on two mitochondrial protein coding genes nicotinamide adenine dinucleotide dehydrogenase subunit 2 (ND2; 1041 bp) and subunit 3 (ND3; 351 bp) published in Reddy and Moyle [17] in order to characterize phylogenetic relationships and divergence times of scimitar babbler species. Since in the Reddy and Moyle [17] study the establishment of species limits was based on multiple samples per taxon, we utilized only a single individual for our phylogenetic analyses to accommodate subsequent niche-based analyses. A total of 29 *Pomatorhinus* taxa were sampled for the ingroup, while our outgroup choice consisted of 3 other babbler species (*Napothera brevicaudata*, *Macronus gularis*, *Garrulax erythrocephalus*).

Phylogenetic relationships within the scimitar babblers were determined using the two mitochondrial protein coding genes [GenBank accession numbers: HQ529038– HQ529235]. Bayesian phylogenetic analysis was carried out within the Markov Chain Monte Carlo (MCMC) tree search algorithm framework as implemented in the program MrBayes 3.1.2 [39]. Since the entire mitochondrial DNA is maternally inherited as a single unit, the two genes were analyzed concatenated and partitioned by each locus and by codon position. We ran two independent runs of 10^7^ generations, using the GTR+I+G model of sequence evolution inferred via the Akaike Information Criterion (AIC) implemented in the program ModelTest 3.7 [40]. Bayesian search parameters included sampling every 100 generations, unlinking of all partition-specific rates and models of evolution, and adjusting of chain heating conditions (temp = 0.5) for improved chain swap acceptance rates. Evaluation of stationarity and chain convergence was conducted by plotting posterior probabilities from the two runs in the program Tracer [41]. The resulting pool of topologies sampled from the first 30% of generations of each of the two independent runs was discarded as an initial ‘burn-in’, and the resulting pool of trees from both runs were summarized to produce a single 50% majority-rule consensus tree, rooted with *Garrulax erythrocephalus*.

To obtain a time calibrated topology for the scimitar babblers, we analyzed the same mitochondrial dataset with BEAST 1.6 [42], using the above-mentioned model of sequence evolution, an uncorrelated lognormal relaxed clock with a Yule speciation parameter, and a broad normally distributed prior on the mutation rate. The mean of the prior distribution was centered around the average coding mitochondrial DNA mutation rate (1.0×10^−8^±3.5×10^−9^ substitution/site/year/lineage) estimated from a diverse array of passerines and based on fossil and biogeographic calibrations [43], [44]. Two runs of 1×10^−8^ generations were performed and the stationary portions of the runs were combined into a single posterior distribution. The tree was again rooted with *Garrulax erythrocephalus*.

### Ecological Niche Modeling and Niche Comparisons

**Figure 3:**
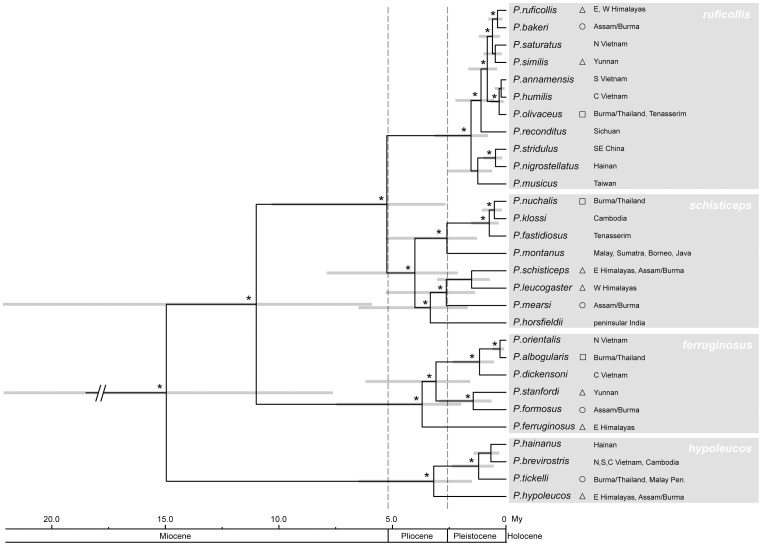
Phylogenetic hypothesis and chronogram of scimitar babbler relationships. Ultrametric time calibrated topology based on the analysis of mitochondrial sequence data from 29 scimitar babbler taxa sampled in the study of Reddy and Moyle [17]. Horizontal grey bars at nodes represent 95% confidence intervals around node heights as generated in the program BEAST, and calibrated using a normally distributed prior on ND2 mutation rates (see Methods). Nodal support above 95% Bayesian posterior probability is indicated by an asterisk. The four main clades are highlighted in grey and labeled with their respective taxonomic designation. Taxa annotated by open triangles occupy Himalayan foothills, while open circles and open squares denote species occupying highlands at lower latitudes (Assam/Burma and Burma/Thailand, respectively).

Scimitar babbler species occurrence data in the form of unique georeferenced localities at which individuals have been recorded via documented records were sourced from specimen vouchers deposited in natural history museum collections throughout the world (see [17]). Ecological variables in the form of 19 bioclimatic GIS layers covering the entire distributional extent of the group were used to summarize aspects of temperature and precipitation from the latter half of the 20th century [45] (http://www.worldclim.org). All GIS layers were clipped to the area of study (Figure 3) and resampled to a spatial resolution of 0.0417 degrees (∼4.5 km).

Ecological niche models [46]–[48] were constructed with MAXENT ver. 3.3.3 [49] using all available environmental layers and occurrence data. Based on an initial set of models ran using all 19 bioclimatic layers, we selected 10 variables that contributed the most towards model performance across all 29 species (Figure 2). While it has been documented that several of the bioclimatic variables can be correlated [50], [51], we were nevertheless interested in examining the disparity and contribution of each variable within the four clades of scimitar babblers given their extensive geographic ranges, heterogeneous habitats, and the fact that we did not project our models onto novel geographic areas or past climatic scenarios. Background points were randomly chosen by the algorithm from within a minimum convex polygon encompassing all occurrence points from all taxa within the four main *Pomatorhinus* clades [13], [52]. For 6 species we had to augment the number of occurrence points to a minimum of 10 spatially unique points (Figure 2). This was done by conducting online searches for recent species reports in the form of published rapid assessment surveys (RAPs) and photographic evidence. Most new observation records usually came from established national forest parks or reserves, which were subsequently verified for habitat integrity and geographic extent through Google Earth imagery. Occurrence points were subset by MAXENT for model testing into training (80%) and testing (20%) data with AUC (Area Under the receiver operating characteristic Curve) values calculated for each run. The cross-validate setting was used for models for species with 10–12 occurrence points (Figure 2), and model averages based on 20 replicate runs per species were used in subsequent analyses. For all species, models were run for a maximum of 5000 iterations and a RAW output was chosen for all further analyses. Niche model outputs were then used to calculate niche overlap based on the *D* and *I* indices as outlined in Warren et al. [53] and implemented in the package ENMTools 1.3 [54]. Both parameters rely on probability distributions over geographic space, and range from 0 (no niche overlap) to 1 (complete niche overlap). Originally intended to reflect relative use of microhabitats or predator-prey interactions, the implementation of the *D* metrics in niche comparisons is not meant to be proportional to any local species densities or habitat use. In comparison, use of the *I* metric does not carry any inherent biological assumptions on the probability of occurrence of a species over geographic space [53], [54].

### History of Predicted Niche Occupancy and Disparity

Analyses of the evolution of niche characteristics usually involve the tracing of species’ tolerance limits inferred from bioclimatic and ecological variables over their phylogenetic history. The majority of studies to date have used either minima and maxima [1], [2], [9] or mean values of ecological variables [10], [11]. Given the discrete nature of these characters traced over evolutionary history, these methods lack the ability to capture the inherent variability and lack of normality of the distribution of ecological variables. As such, recently proposed methods that extract a niche occupancy profile of each environmental variable from a species’ ecological niche model [12], [13] aim to capture and quantify the inherent multimodal variation in ecological parameter space over the geographic range of a species. Here, we follow this methodology to extract, produce and analyze predicted niche occupancy (PNO) profiles in a phylogenetic context.

First, in order to produce PNO profiles, each bioclimatic layer used in the ecological niche modeling algorithm is integrated over every species’ MAXENT probability distribution. The resulting distribution characterizes the PNO profiles in unit area histograms of suitability for each species’ predicted occupancy of each bioclimatic variable [12], [13]. Our approach to obtain PNO profiles was to use RAW output values from the MAXENT probability distributions of each species and divide these into 50 equally spaced bins spanning the parameter range of each bioclimatic variable.

After extracting all PNO profiles for each of the scimitar babblers, we used these distributions to investigate aspects of phylogenetic evolution of niche occupancy. For this we calculated the maximum likelihood estimate of each bioclimatic variable at each internal node of the time-calibrated species phylogeny under the assumption of a Brownian model of evolution. We performed 100 replicate draws of values from each of the species’ PNO profiles, thus sampling more effectively the inherent variation of each bioclimatic distribution. The resulting plots of histories of niche evolution convey information on the directionality of niche evolution by illustrating convergent evolution (among clades) as well as divergent evolution (within clades). These analyses were performed with the R packages PHYLOCLIM and PHYLOCH, available through the Comprehensive R Archive Network repository (http://cran.r-project.org/web/packages) or directly from the author’s website (http://www.christophheibl.de/Rpackages).

Next, values obtained through PNO plots were used in the calculation of relative disparity through time (DTT) [55]. This analysis contrasts the time course of climatic niche overlap within and among clades [12]. It is based on the calculation of average pairwise Euclidean distance between clade members divided by the disparity of the entire phylogenetic tree. On the horizontal axis, DTT plots are constrained to range from 0 at the root of the topology to 1 at the tips (present day) of the time-calibrated tree. Although disparity values on the vertical axis are also constrained to range between 0 and 1, observed values can exceed values of 1 in cases in which disparity within subclades at a given node is especially large. Observed values along the DTT plot closer to 0 indicate that respective internodes contain relatively little of the variance present within the clades, thus also denoting divergence in characters. Observed nodal values closer or above 1 indicate that substantial amount of variance is clustered within clades, denoting conservatism or convergent evolution towards similar parameter space of ecological characters [12], [13], [55]. In order to quantify the disparity between our observed data and an unconstrained null model of Brownian motion evolution of ecological characters based on 1000 simulations of each ecological and topographic variable, we calculated the Morphological Disparity Index (MDI) [55]. MDI represents the difference between observed disparity and the disparity simulated under the null model and is derived as the area contained between the line connecting observed relative disparity points versus the line connecting median relative disparity points of the null model. Positive MDI values indicate that disparity is distributed mostly within subclades due to niche evolution within subclades, while negative MDI values suggest disparity is distributed mostly among subclades due to niche conservatism within subclades. DTT plots and calculations of MDI were performed through the R package GEIGER [56] (http://cran.r-project.org/web/packages). We produced DTT plots taking into account the entire *Pomatorhinus* phylogeny as well as for individual clades, for which we present only the MDI values. Clade based DTT analyses were intended to show additional variation of the accumulation of disparity relative to the different cladogenetic time frames of each of the four *Pomatorhinus* groups (Figure 3).

## Results

### Phylogenetic Relationships and Ecological Niche Models of Pomatorhinus Babblers

Using a subset of their data, a total of 29 ingroup taxa and 1557 molecular characters, our Bayesian reconstruction recovered the same phylogenetic structure comprising four main *Pomatorhinus* clades as published in Reddy and Moyle [17]. The *ruficollis* clade contains 11 species, the *schisticeps* clade contains 8 species, the *ferruginosus* clade contains 6 species, while the *hypoleucos* clade contains only 4 species. Overall, our chronology of cladogenesis based on mutation rate priors [43], [44] lies within the time frames outlined by Reddy and Moyle [17]. As expected, the *ruficollis* clade originated comparatively recently in the late Pleistocene, while the remaining 3 clades had an early Pleistocene origin (Figure 3). Relationships among major *Pomatorhinus* scimitar babbler clades estimated using the present mitochondrial dataset [17] are in agreement with results published in a recent multilocus systematic revision of core babblers [57].

Ecological niche models were in general accordance with presently known range maps [17], [25]. Highest species diversity was observed in the Himalayan foothills region of NE India, where up to 14 species in all four clades are predicted to occur in sympatry (Figure 1). Values of the AUC ranged from 0.89 to 1, indicating excellent model performance (Figure 2). The contribution of the 10 bioclimatic variables to the ecological models ranged from 0 to 89.5% (Figure 2). While there was no clear trend in certain variable preferences between species, we did observe that Temperature Seasonality and Precipitation of the Warmest Quarter generally contributed most to the models across all species. Niche models illustrating the potential distribution of individual *Pomatorhinus* species in the form of binary suitability maps with minimum training presence threshold applied are presented in the supplementary materials (Figure S1).

**Figure 4:**
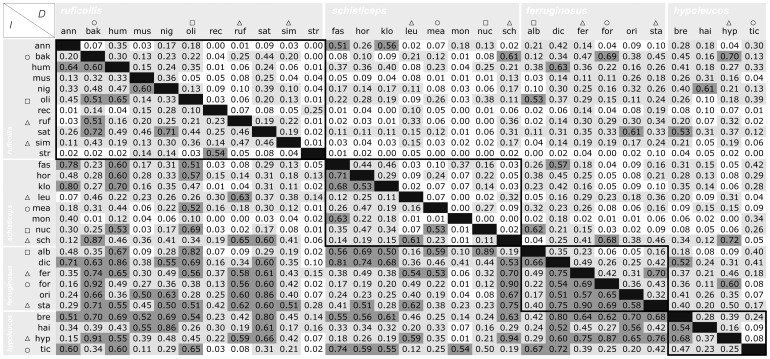
Niche overlap values between and within clades of *Pomatorinus* babblers. Summary of geographic niche overlap values as calculated by the *D* (above diagonal) and *I* (below diagonal) statistics as outlined in Warren et al. [49]. Species are grouped by clades identified through the phylogenetic analysis outlined in Figure 3. Species abbreviations reflect the first three letters of their specific epithet, and taxa annotated by open triangles occupy Himalayan foothills, while open circles and open squares denote species occupying highlands at lower latitudes (Assam/Burma and Burma/Thailand, respectively). Boxed values represent overlap comparisons between taxa within their respective clades. Overlap values ranging from 0.11–0.50 are highlighted in light grey, while values above 0.50 are highlighted in dark grey.

Comparisons of niche overlap within and among clades (Figure 4) indicate lower overlap within the *ruficollis* clade, while the *ferruginosus* clade had higher overlap values between its constituent taxa. Highest niche overlap values were recorded between two taxa from different clades occupying the Himalayan region (open triangle, Figure 4), where *P. schisticeps* (*schisticeps* clade) and *P. hypoleucos* (*hypoleucos* clade) had *D* and *I* indices of 0.70/0.94, respectively. Overlap in geographic niche space between taxa in the Himalayan foothills (open triangles, Figure 4) was generally at or above the 0.5 threshold value for *I*, indicating sympatric distributions in their predicted ecological niches. Within a clade, niche overlap indices observed between two Himalayan species were highest (0.70/0.90, respectively) in the *ferruginosus* clade, for *P. ferruginosus* and *P. stanfordi*. Niche overlap between the two species endemic to Hainan Island, *P. nigrostellatus* (*ruficollis* clade) and *P. hainanus* (*hypoleucos* clade) were also very high, with values of 0.61/0.86 for *I* and *D*, respectively.

### Phylogenetic History of Niche Evolution

**Figure 5:**
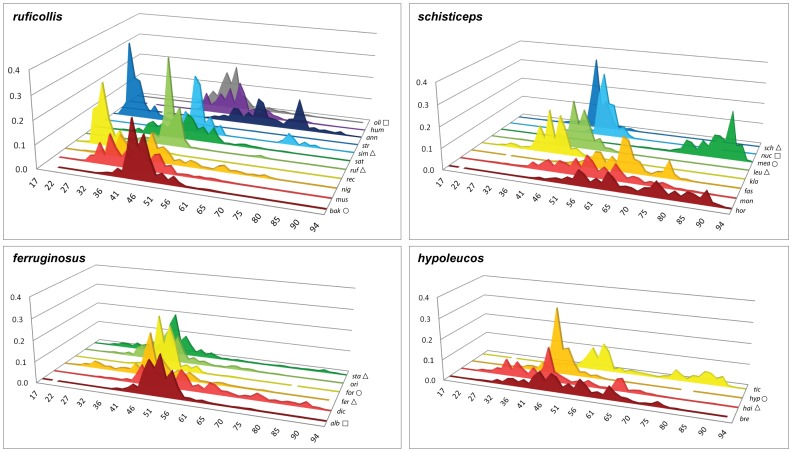
Predicted niche occupancy (PNO) profiles for the four clades of *Pomatorhinus* scimitar babblers. Horizontal axes represent the Isothermality (Bio3 - the tolerance of diurnal temperature range divided by the annual temperature range, all multiplied by 100) parameter space divided into 50 equally spaced bins, while vertical axes denote the total suitability of the isothermality index of each species over its entire geographic range (see Methods). Specific epithets have been reduced to three letter codes for brevity. Taxa annotated by open triangles occupy Himalayan foothills, while open circles and open squares denote species occupying highlands at lower latitudes (Assam/Burma and Burma/Thailand, respectively). Overlapping peaks of PNO profiles indicate similar climatic tolerances, while the overall breadth of the profile denotes the degree of specificity in climatic tolerance. See also Figure S2 for PNO graphs of all 10 bioclimatic variables used in the niche modeling algorithm of the 4 *Pomatorhinus* clades and their constituent species.

Rather than relying on single estimates of means or centroids of ecological variables, our results based on predicted niche occupancy (PNO) profiles derived from MAXENT predictions illustrate marked heterogeneity of histograms of each bioclimatic variable across clades (Figure 5). This heterogeneity is further accentuated when all bioclimatic variables are compared within each clade (see Figure S2). For example, in case of the Isothermality index it is noteworthy that members of the *ruficollis* group occupy different regions of the parameter space, such that *P. bakeri*, *P. ruficollis*, *P. similis*, and *P. olivaceus* all have largely overlapping distributions of similar shape, while the remaining member species occupy regions to the left and right of the parameter space (Figure 5). This pattern is indicative of adaptation to different temperature range tolerances as well as niche partitioning within this clade. As such, *P. reconditus* and *P. stridulus* can only tolerate diurnal temperature ranges of about 25% of its total annual temperature range within the geographic extent of our study. A similar heterogeneity can also be observed for members of the *schisticeps* group, where *P. montanus* (the only species to occur in the Sunda islands) shows the highest Isothermality tolerance, while members of the *ferruginosus* and *hypoleucos* clades appear to have converged to occupy similar Isothermality parameter ranges. Overall, PNO profiles of temperature and precipitation variables tended to cluster taxa in similar parameter spaces (Figure S1). Scimitar babblers were predicted to tolerate the lower spectrum of the Temperature Annual Range (Figure S2; 9.6–33.0°C) and lower spectra of parameters linked to Precipitation (Figure S2; >3000 mm).

**Figure 6:**
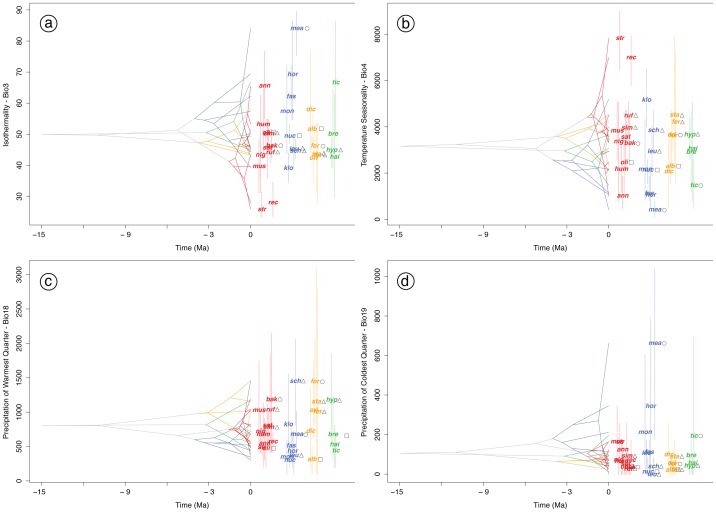
History of evolution of climatic tolerances *Pomatorhinus* scimitar babblers. The maximum a posteriori chronogram topology produced by the BEAST analysis and the PNO profiles of four ecological variables have been integrated to represent the reconstruction of mean climatic tolerances based on 100 random samples of the PNO profiles at internal nodes. Panel (a) depicts the evolution of Isothermality (Bio3), (b) reconstructs Temperature Seasonality (Bio4), while (c) summarized the evolution of Precipitation of the warmest quarter (Bio18), and (d) of Precipitation of the coldest quarter (Bio19). Members of the four main *Pomatorhinus* clades are colored red (*ruficollis*), blue (*schisticeps*), yellow (*ferruginosus*), and green (*hypoleucos*). The three letter codes abbreviating the specific epithets of each species are positioned at the mean of the 80% central density. Taxa occupying Himalayan foothills are denoted by open triangles, Assam/Burma highlands by open circles, and Burma/Thailand highlands are represented by open squares. Overlapping internal nodes indicate convergent climatic origins, while crossing branches of the phylogenetic tree indicate convergent niche evolution among taxa from different clades. See also Figure S3 for plots of evolution of climatic tolerances of all 10 bioclimatic variables used in niche modeling algorithm of the present study.

By analyzing the PNO profiles in a phylogenetic context it is evident that the *Pomatorhinus* scimitar babblers have radiated extensively over a broad spectrum of the ecological space analyzed (Figure 6). In the aforementioned example of the Isothermality variable (Figure 6a) as well as the Temperature Seasonality (Figure 6b), we can observe a divergent evolution within a broad spectrum of the parameter space in all members of the four clades. Specifically, members of the *ruficollis* and *schisticeps* clades occupy both low and high ranges of values in both of these variables, with a few species clearly having diverged to separate regions within the parameter space. Such is the case of *P. stridulus* and *P. reconditus* (*ruficollis* group), and *P. klossi* and *P. mearsi* (*schisticeps group*). Overall, divergent evolution of values for these two bioclimatic variables can be deduced by the general lack of overlapping branches leading to the mean climatic tolerances of each species (Figure 6a, Figure 6b). In contrast to this pattern are the bioclimatic variables pertaining to Precipitation of the Warmest (Bio 18; Figure 6c) and Coldest Quarters (Bio 19; Figure 6d). Here we observed a convergent evolution of species from different clades (*P. schisticeps* - *schisticeps* clade with *P. formosus* - *ferruginosus* clade, *P. bakeri* - *ruficollis* clade, with *P. stanfordi - ferruginosus* clade and *P. hypoleucos* - *hypoleucos* clade) towards similar portions of the parameter space (Figure 6c). Taxa occupying Himalayan foothills (open triangles, Figure 6b) were inferred to tolerate higher amounts of Temperature Seasonality than taxa distributed further south in the Assam/Burma and Burma/Thailand regions (open circles and open squares, respectively; Figure 6b). In terms of Precipitation of the Warmest Quarter (Figure 6c), mean tolerances of Himalayan taxa of the *ruficollis* and *ferruginosus* clades (*P. ruficollis* + *P. similis* and *P. stanfordi* + *P. ferruginosus*, respectively) were clustered between that of more southern taxa from Assam/Burma and Burma/Thailand (*P. bakeri* + *P. olivaceus* and *P. formosus* + *P. albogularis*, respectively). This trend is not repeated in the *schisticeps* and *hypoleucos* clades, where Himalayan taxa (*P. schisticeps* + *P. leucogaster* and *P. hypoleucos*, respectively) are situated in regions of higher precipitation regimes than species from the same clades distributed further south (*P. nuchalis* + *P. mearsi* and *P. tickelli*, respectively). The evolution of predicted niche occupancy profiles of all climatic and topographic variables is further detailed in the supplementary materials (Figure S3).

**Figure 7:**
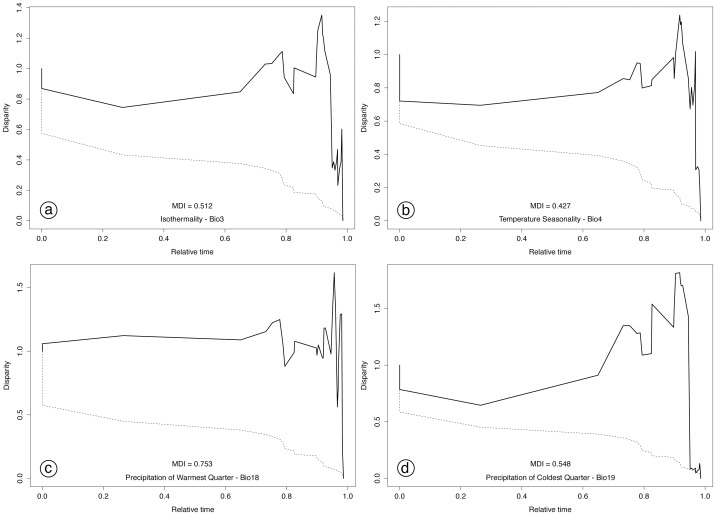
Plots of accumulation of relative ecological disparity through time (DTT). Plot summarizes all 29 Southeast Asian *Pomatorhinus* scimitar babblers (solid line) compared with mean disparity as simulated under 1000 replicates of an unconstrained model of Brownian Evolution (dashed line). Disparity plots start out in the left side or the graph (root of topology) at a value of 1 and end on the right (all extant taxa) at a value of 0. Disparity represents the mean of the square pairwise differences between all terminal taxa defined at each node (see Methods). The same four bioclimatic variables as in Figure 6 (Isothermality, Temperature Seasonality, Precipitation of Warmest Quarter, Precipitation of Coldest Quarter) are represented in distinct panels. The morphological disparity index (MDI) value represent the overall difference in disparity between the observed and the unconstrained null hypothesis.

**Figure 8:**
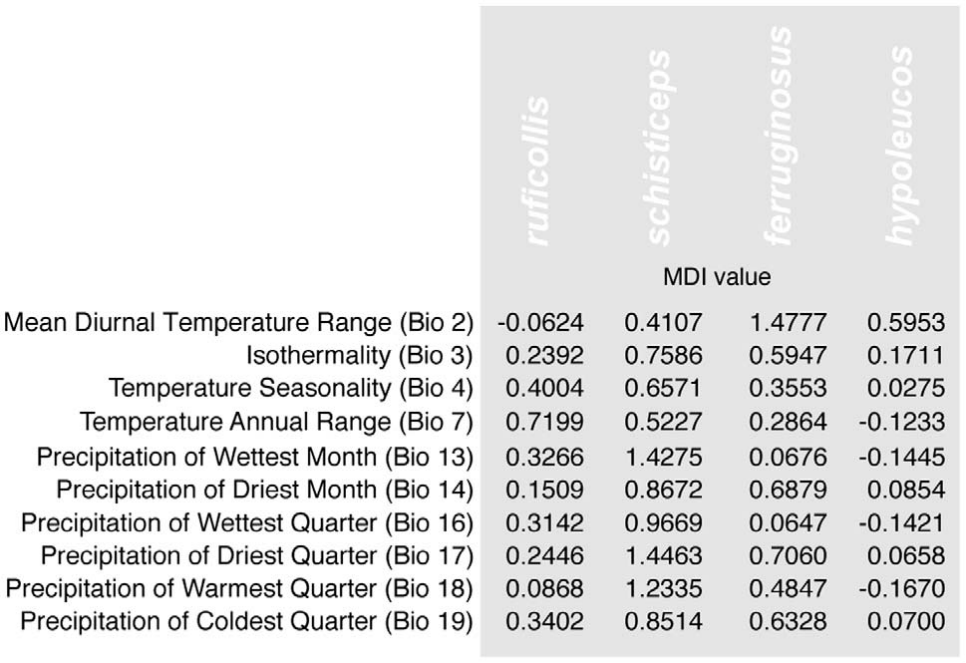
Clade-based calculations of MDI values. Comparison of the morphological disparity index (MDI) derived through the analysis of disparity through time (DTT) plots of individual clades. The MDI values represent the overall difference in disparity between the observed and the unconstrained null hypothesis (see Methods and Figure 7). Values of MDI below zero are indicate that disparity tends to be distributed among subclades, while MDI values above zero indicate that disparity tends to be distributed within subclades.

Accumulation of ecological disparity analyzed through DTT plots indicates a departure of observed patterns from the Brownian model of evolution (Figure 7). In most cases within the *Pomatorhinus* radiation, ecological disparity starts at relatively high values (about 0.7) early on, and tends to accumulate even more, reaching values above 1.5 at later stages in their evolutionary history. In addition to this pattern, we can infer a relatively stable progression of disparity at early time frames. This is illustrated by the lack of variation in the progression of the line of observed relative disparity from timeframe 0 (base of the topology) towards the recent times (tips of the topology), not overlapping with the null model of evolution (Figure 7, dotted line). Using the previously highlighted examples of the four variables based on measures of temperature (Isothermality and Temperature Seasonality) and precipitation (of Warmest Quarter and Coldest Quarter), it is apparent that overall accumulation of disparity within subclades increased towards recent timeframes. For the phylogeny based analysis examining disparity across clades (Figure 7, Figure S4), all values of MDI were positive (Figure 7, Figure S4), indicating that overall disparity is distributed within subclades rather than among them. In case of the clade based analysis (Figure 8), values of MDI were all positive for species of clades A, B and C, whereas in clade D we observed negative MDI values for most variables.

## Discussion

To our knowledge this represents the first avian study to explore the evolution of multidimensional ecological characters over phylogenetic history in a diverse radiation within Southeast Asia. This integration can shed further light onto the complex biogeographic history of this dynamic group. Nodes uniting the four *Pomatorhinus* clades extend more distantly into the Miocene, but major speciation events within these groups occurred in the Plio-Pleistocene [17] (Figure 3). As a consequence of this rapid burst of speciation, scimitar babblers have had the opportunity to diversify over a broad geographic and ecological range within Southern Asia (Figure 1; Figure S1). However, variability in spatio-temporal aspects of these divergences highlights similarities and differences in the patterns of niche evolution of these species.

Our analysis of the ecological niches based on 10 most informative climatic variables (Figure 2) indicate highest overlap of potential distributions within the foothills of the Himalayas, in the area of NE India and N Vietnam and SW China. Another geographic region predicted to harbor ecological parameters that would support high scimitar babbler species diversity lies along the western coast of India. Although only *P. horsfieldi* presently has populations in this area, the fact that other *Pomatorhinus* species were predicted to occur in this region is a representation of the extent of the potential ecological niche over disjunct geographic space [58], [59]. Areas where only few species were predicted to occur, such as in central and SE China (*P. stridulus* and *P. reconditus*) or the Sunda Islands (*P. montanus*) generally occupy more peripheral ecological parameter space, suggesting departure from ecological optima for high species diversity.

Generally, scimitar babblers occupy warmer and humid spectra of the Mean Annual Temperature and Annual Precipitation variables (Figure S2). Evolution of the 10 ecological parameters analyzed within a phylogenetic framework indicated a signature of both divergent and convergent evolution (Figure 6, Figure S3). Specifically, *P. stridulus* and *P. reconditus* are two of the most ecologically divergent taxa within the *ruficollis* clade, occupying more extreme value ranges in case of Isothermality, Temperature Seasonality, and Temperature Annual Range (Figure 6, Figure S3). Although not sister species, both of these taxa have very similar morphologies but occur in different forested habitats in southeastern and southcentral China. The climatic variables delineating these species are thus indicative of different tolerances to temperature fluctuations compared to the remaining members of their clade. Convergent evolution has been also observed for most variables and at varying temporal scales. We observed marked convergence to similar mean variable ranges also among internal nodes of the four *Pomatorhinus* clades (Figure 6c, Figure 6d). In the case of Precipitation of the Warmest Quarter it is noteworthy that species from three of the four *Pomatorhinus* clades converged onto virtually the same mean value of around 1400 mm precipitation (Figure 6c). These species (*P. bakeri*, *P. stanfordi*, and *P. hypoleucos*) are found at higher elevations in the Himalayas and the adjacent Burma/Thailand highlands. Not surprisingly, the sympatrically distributed *P. bakeri* (*ruficollis* clade) and *P. hypoleucos* (*hypoleucos* clade) also had among the highest values of geographic overlap of their potential ecological niches (see Results and Figure 4). An even more striking convergence could be observed in the case of the Precipitation of the Wettest Month and Wettest Quarter, where members of all four *Pomatorhinus* clades (*P. bakeri, P. schisticeps, P. formosus, P. hypoleucos*) converged virtually on the same mean value of around 650 mm and 1500 mm, respectively (see Figure S3).

Examination of the evolution of predicted niche occupancy (PNO) profiles further illustrates the usefulness of incorporating a phylogenetic hypothesis in form of a chronogram to map the positions of reconstructed internal nodes in ecological parameter space and relative divergence time. Here it is immediately apparent that the rapid speciation burst witnessed by the 11 species of the *ruficollis* clade is in fact the youngest of all *Pomatorhinus* clades, and at the same time also among the most ecologically diverse (Figure 6, Figure S3). With few exceptions, species of the *ruficollis* clade occupy the same or a larger breadth of ecological parameter space than clades that have speciated at earlier time frames. Generally we observed a trend towards divergent evolution within clades and convergent evolution among clades, illustrated by the overlapping branches of different clades. This fact highlights the profound influence of rapid diversifications witnessed by continental Southeast Asian taxa, most likely attributed to climatic fluctuations and orogenic events of the Plio-Pleistocene [60]–[62].

Looking at the evolution of niche characteristics between recently diverged scimitar babbler sister species, such as the *P. schisticeps* – *P. leucogaster* pair from the *schisticeps* clade, it is evident that although these two taxa presently occupy Himalayan foothill habitats, they show rather distinct preferences in their ecological parameter space as identified by both PNO plots (Figure 5; Figure S2) as well as in the evolution of their niche occupancy profiles (Figure 6; Figure S3). This indicates significant distinctions in habitat between the western and eastern Himalayas, perhaps explaining why species numbers in these two areas are so different (see [27]). Interestingly, there appears to be less ecological differentiation between species from the Eastern Himalayas and adjacent highlands such as Yunnan and Assam/Burma (e.g. *P. ruficollis* and *P. bakeri* in the *ruficollis* clade, and *P. stanfordi* and *P. formosus* in the *ferruginosus* clade). This relatively low level of niche differentiation can potentially be attributed to a more recent speciation in geographically adjacent habitats that inherently harbor less heterogeneity in broad scale climatic variables. At the same time, this pattern is also suggestive of niche conservatism over shorter periods of time [32].

Studies involving the use of environmental niche-based reconstructions to project species distributions into past climatic scenarios are faced with the problem of potentially encountering non-equivalent climatic conditions at diverse time frames [63], [64]. While these studies rely on retro-projecting niche-based conditions onto past geographies with the aim of identifying potential stable refugia or areas of suitable conditions for dispersal, phyloclimatic studies are relying on niche-based characters to infer the overall direction and pattern of current climatic tolerances of taxa, and do not necessarily require explicit geographic projections of past climate conditions.

Disparity through time plots (DTT) further accentuate the signature of rapid accumulation of ecological diversity within recent time frames. Relative disparity emerges rapidly within *Pomatorhinus* scimitar babblers, identified by disparity values over 0.7. Early disparity values are mostly following a steady course (Figure 7, Figure S4). This trend continues past the relative time of common ancestors within the Miocene (corresponding to the first three perceptible dips in the solid line) and becomes increasingly more rugged towards the Pliocene and Pleistocene (around relative time frame of 0.8 on the horizontal axis of the DTT plots). All MDI values were positive, indicating significant departure of our observed disparity trends from the null model simulated under a Brownian motion mode of evolution [12], [13], [55]. This also indicates the fact that the accumulation of disparity tends to be distributed within subclades rather than among subclades. This general trend of accumulation of disparity through time contrasts well with previous studies of niche evolution in plants [12], [13], where deeper divergences cause the DTT plots to show generally less accumulation of disparity within clades at earlier time frames. Again, we believe that the rapid diversification of scimitar babblers within the Plio-Pleistocene due to intense orogenic and climatic influences has resulted in early shifts of taxa towards new climatic niches and geographic distributions.

Calculations of MDI values based on DTT plots for individual clades highlights some shared patterns of the distribution of disparity within subclades. The *ruficollis, schisticeps*, and also the *ferruginosus* clades had mostly positive MDI values, suggesting that due to niche evolution, disparity is again distributed predominantly within rather than among constituent subgroups in these clades. Only the *hypoleucos* clade featured mostly negative MDI values due to niche conservatism and disparity being distributed among subclades. This pattern was expected due to the low number of constituent member species and lack of intra-clade substructure (Figure 3). Comparing the two most speciose and biogeographically similar clades, the *ruficollis* (11 species) and *schisticeps* (8 species), it becomes apparent that all except one of the MDI values were positive, pointing towards considerable niche evolution in these clades. One key difference was observed in the magnitude of MDI values for 3 precipitation variables, for which the *ruficollis* clade had much lower MDI values compared to the *schisticeps* clade (Figure 8). This points towards a more significant within-clade accumulation of disparity in the case of the *schisticeps* clade.

Climatic tolerances of species occurring in the Sino-Himalayan range also indicate convergence towards similar ecological parameter spaces in non-sister species within clades (*P. ruficollis* – *P. similis*, *ruficollis* clade; *P. stanfordi* – *P. ferruginosus*, *ferruginosus* clade) but also among species from different clades (*P. ruficollis* – *P. stanfordi – P. hypoleucos*). On the other hand the single instance of an extant Himalayan sister species pair (*P. schisticeps* – *P. leucogaster*, *schisticeps* clade) has resulted in the divergence into quite different ecological space, at least as illustrated by the Precipitation of Warmest Quarter parameter (Figure 6c). These two species, however, occupy Eastern and Western Himalayan ranges, respectively, where the Eastern region harbors drier conditions that the Western part of the range. Therefore, we believe that the biogeographic and phylogenetic origins of Himalyan taxa at different temporal scales have led to ecological differentiation only in recently diverged taxa.

In this study, we highlight the clear utility of this suite of methods for its increased explanatory power in biogeography by integrating ecological and phylogenetic analyses. We identified distinct evolutionary trends in ecological parameters used in niche modeling algorithms, both in terms of convergent and divergent niche tolerances in related species. Phyloclimatic analyses, therefore, are especially suitable for the study of niche evolution in groups containing numerous codistributed lineages. While our study focuses on documenting the evolution of climatic tolerances within and among distinct clades, we do recognize the need for further studies that account and test for alternative scenarios of modes and rates of evolution of these traits, something that has been explored recently by means of comparing how different models of trait variances in a time-calibrated phylogeny are distributed within and among subclades [65].

## Supporting Information

Figure S1 **Ecological niche models of 29 Southeast Asian** ***Pomatorhinus*** **scimitar babblers.** Models are based on MAXENT thresholded (minimum training presence) binary outputs. Areas in red represent regions of predicted to support populations based on 10 bioclimatic features with highest model contribution across all 29 scimitar babbler species. Species are grouped by clades as defined by the phylogenetic hypothesis in Figure 3.
(TIF)

Figure S2 **Predicted niche occupancy plots (PNO) for the four clades (panels A, B, C, D) of** ***Pomatorhinus*** **scimitar babblers.** Color scheme follows Figure 5. Bioclimatic layers used in the MAXENT modeling algorithm are listed by their names and abbreviations as outlined in Figure 2. Species abbreviations have been omitted. Vertical axes represent cumulative unit are of suitability, while the different horizontal axes represent the entire parameter space of each variable divided into 50 equally spaced bins (see Methods).
(TIF)

Figure S3 **Plots summarizing the evolution of climatic tolerances in** ***Pomatorhinus*** **scimitar babblers.** Plots are based on PNO profiles for 10 bioclimatic layers with highest model contribution used in the MAXENT modeling algorithm. Abbreviations for each of these variables are given in Figure 2. Colors denote different clades and follow the same scheme used in Figure 6. Species abbreviations use 3 letter codes as indicated in Figure 6.
(TIF)

Figure S4 **Disparity through time plots (DTT) of all** ***Pomatorhinus*** **scimitar babblers.** Plots depict the 10 bioclimatic layers with highest model contribution used in the MAXENT modeling algorithm, and include the entire 29 species of *Pomatorhinus* scimitar babblers. Vertical axes represent disparity, while horizontal axes depict evolutionary time. Observed values (solid line) are compared with mean disparity as simulated under 1000 replicates of an unconstrained model of Brownian Evolution (dashed line). Disparity plots start out in the left side or the graph (root of topology) at a value of 1 and end on the right (all extant taxa) at a value of 0. Disparity represents the mean of the square pairwise differences between all terminal taxa defined at each node (see Methods). The morphological disparity index (MDI) value represent the overall difference in disparity between the observed and the unconstrained null hypothesis.
(TIF)
