# Supplementary figures and images for: Comparative Phyloclimatic Analysis and Evolution of Ecological Niches in the Scimitar Babblers (Aves: Timaliidae: Pomatorhinus)
Source: PLoS One. 2013 Feb 6;8(2):e55629. doi: 10.1371/journal.pone.0055629 (PMC3566057; doi:10.1371/journal.pone.0055629)

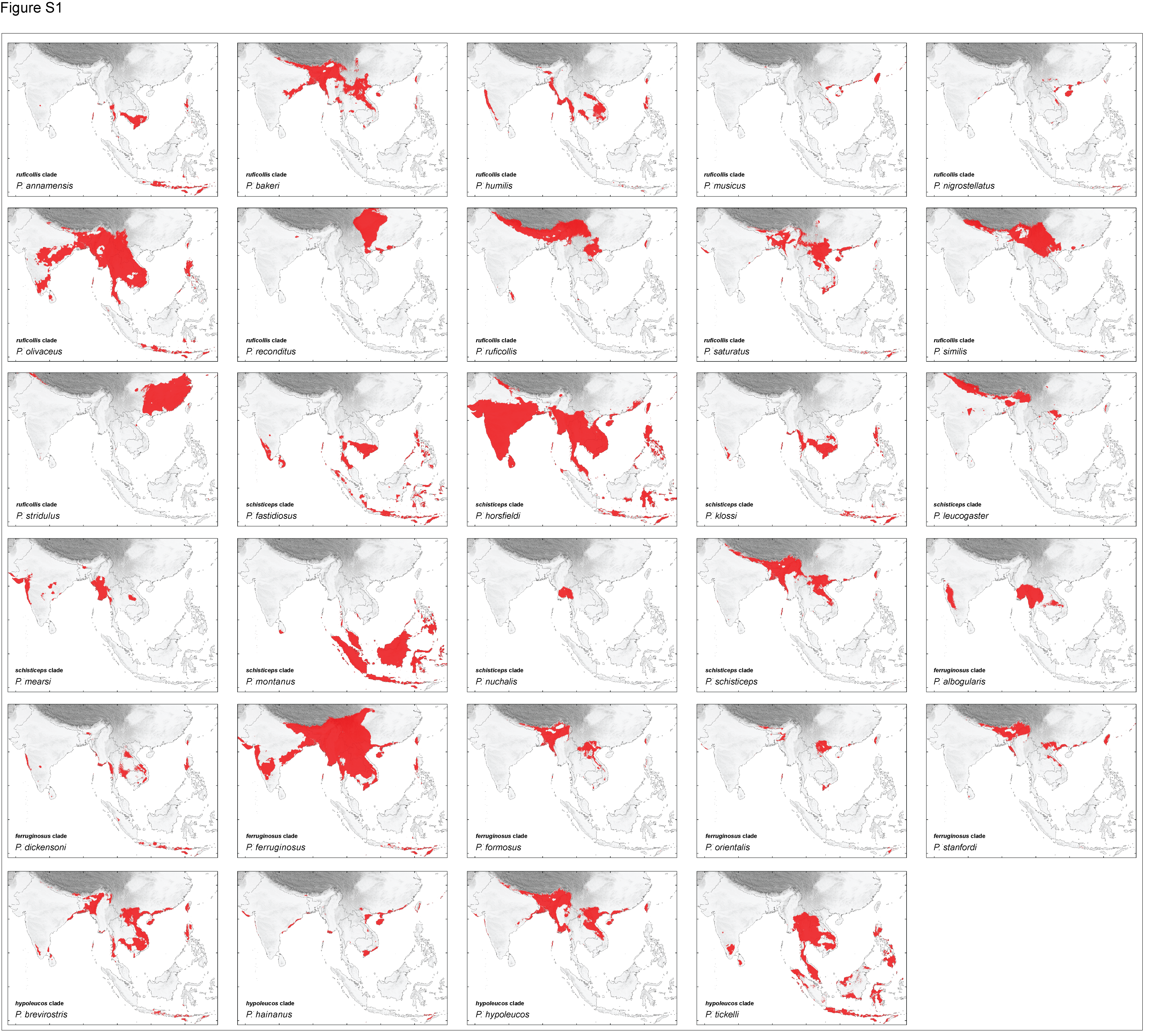

Supplement: Figure S1 — Ecological niche models of 29 Southeast Asian Pomatorhinus scimitar babblers. Models are based on MAXENT thresholded (minimum training presence) binary outputs. Areas in red represent regions of predicted to support populations based on 10 bioclimatic features with highest model contribution across all 29 scimitar babbler species. Species are grouped by clades as defined by the phylogenetic hypothesis in Figure 3. (TIF) [file pone.0055629.s001.tif]

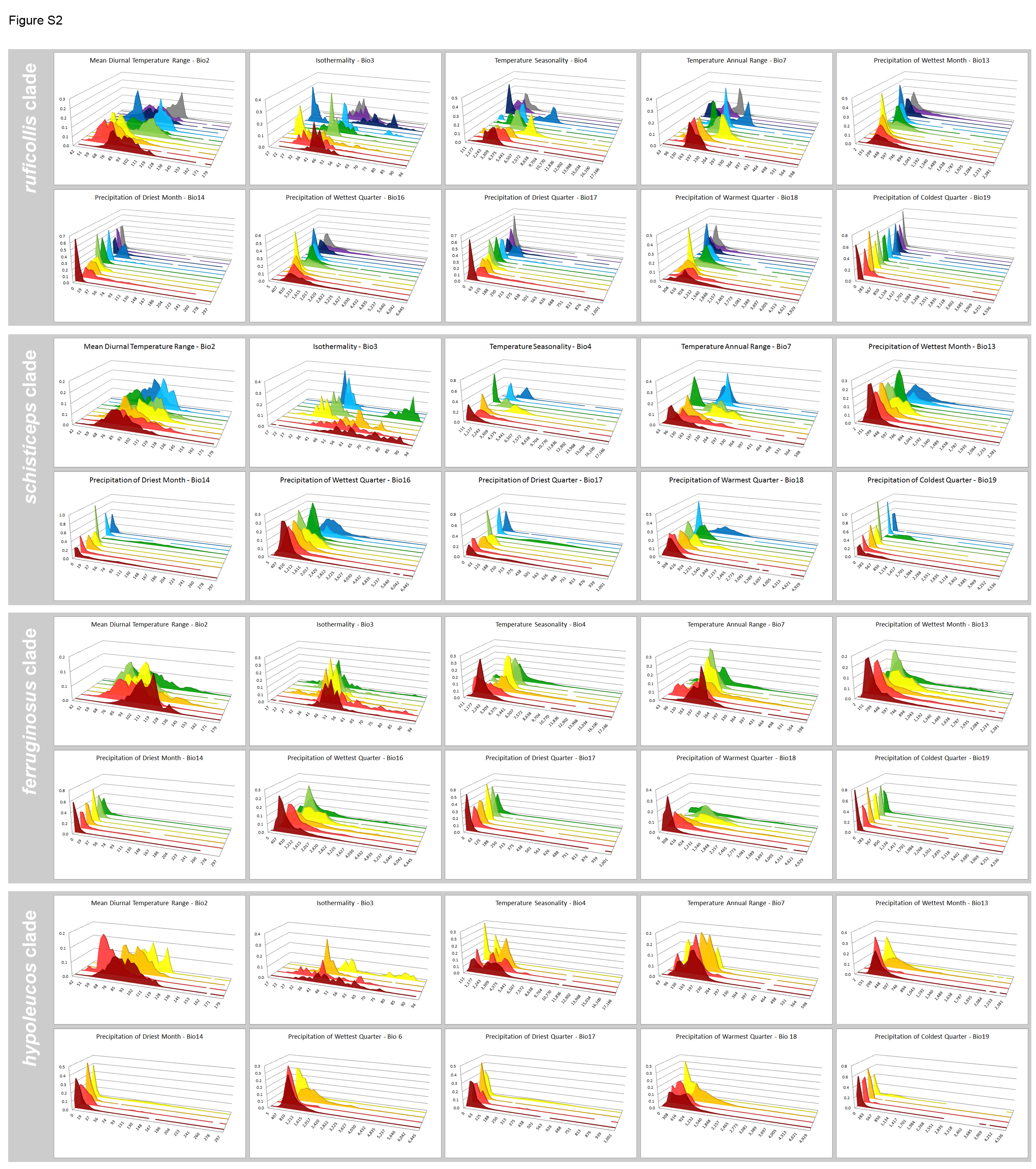

Supplement: Figure S2 — Predicted niche occupancy plots (PNO) for the four clades (panels A, B, C, D) of Pomatorhinus scimitar babblers. Color scheme follows Figure 5. Bioclimatic layers used in the MAXENT modeling algorithm are listed by their names and abbreviations as outlined in Figure 2. Species abbreviations have been omitted. Vertical axes represent cumulative unit are of suitability, while the different horizontal axes represent the entire parameter space of each variable divided into 50 equally spaced bins (see Methods). (TIF) [file pone.0055629.s002.tif]

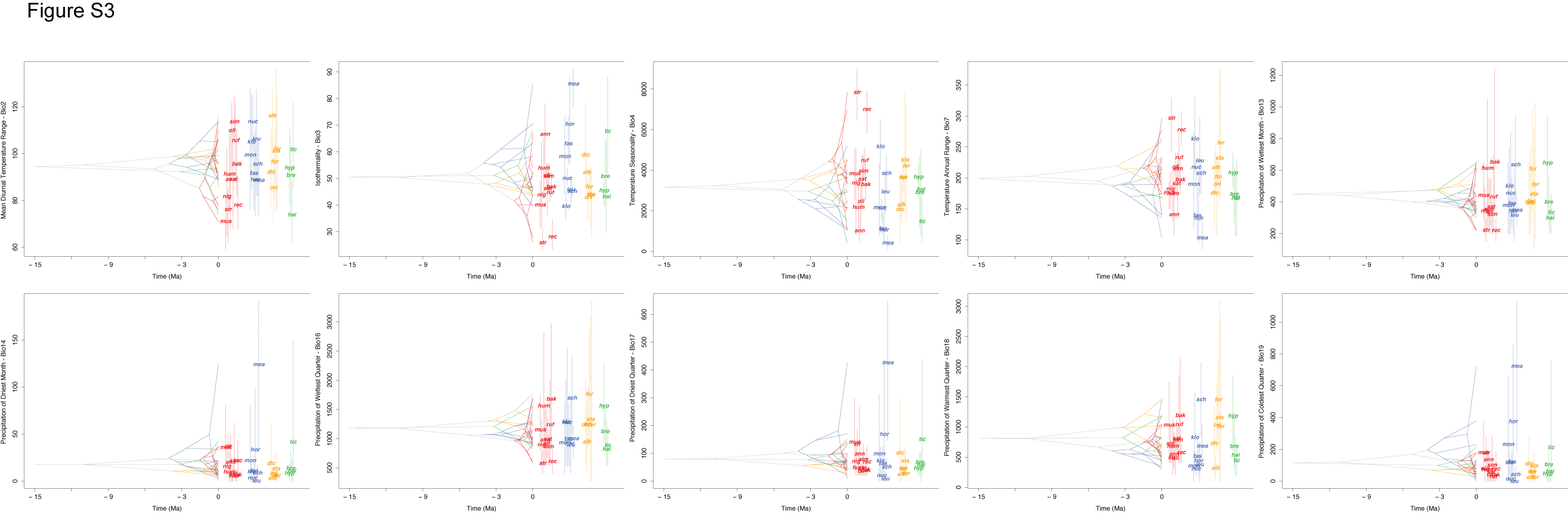

Supplement: Figure S3 — Plots summarizing the evolution of climatic tolerances in Pomatorhinus scimitar babblers. Plots are based on PNO profiles for 10 bioclimatic layers with highest model contribution used in the MAXENT modeling algorithm. Abbreviations for each of these variables are given in Figure 2. Colors denote different clades and follow the same scheme used in Figure 6. Species abbreviations use 3 letter codes as indicated in Figure 6. (TIF) [file pone.0055629.s003.tif]

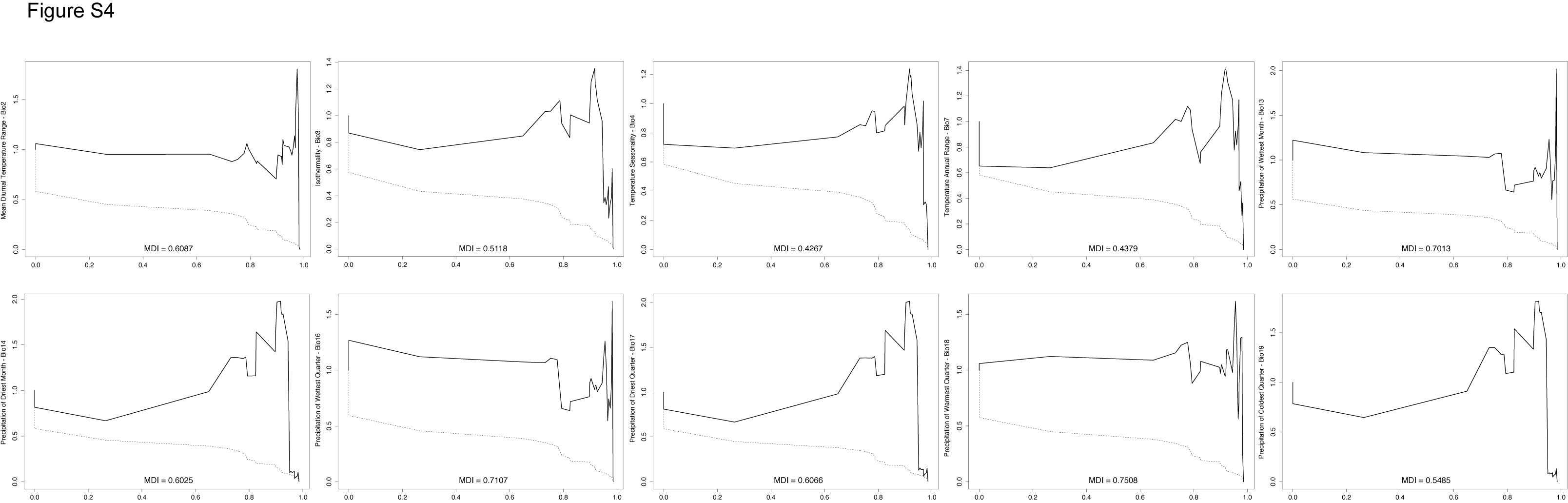

Supplement: Figure S4 — Disparity through time plots (DTT) of all Pomatorhinus scimitar babblers. Plots depict the 10 bioclimatic layers with highest model contribution used in the MAXENT modeling algorithm, and include the entire 29 species of Pomatorhinus scimitar babblers. Vertical axes represent disparity, while horizontal axes depict evolutionary time. Observed values (solid line) are compared with mean disparity as simulated under 1000 replicates of an unconstrained model of Brownian Evolution (dashed line). Disparity plots start out in the left side or the graph (root of topology) at a value of 1 and end on the right (all extant taxa) at a value of 0. Disparity represents the mean of the square pairwise differences between all terminal taxa defined at each node (see Methods). The morphological disparity index (MDI) value represent the overall difference in disparity between the observed and the unconstrained null hypothesis. (TIF) [file pone.0055629.s004.tif]
